# Marker Screw Utilization for Minimally Invasive Transforaminal Lumbar Interbody Fusion (MS-MIS TLIF): Promises and Advantages

**DOI:** 10.3390/medicina59030585

**Published:** 2023-03-16

**Authors:** Mohammed Khashab, Moyassar Karami, Muath Alswat, Mohamed Elkhalifa

**Affiliations:** 1 Department Surgery, Orthopaedic Division, Ministry of National Guard—Health Affairs, Jeddah 22384, Saudi Arabia; 2 King Abdullah International Medical Research Center, Jeddah 22384, Saudi Arabia; 3 College of Medicine, King Saud Bin Abdulaziz University for Health Sciences, Jeddah 22384, Saudi Arabia

**Keywords:** MIS TLIF, pedicle screw, marker screw, guidance methods, fusion, degenerative lumbar disease

## Abstract

*Background and Objective*: Minimally Invasive Transforaminal Lumbar Interbody Fusion (MIS-TLIF) has been investigated and shown excellent short- and long-term outcomes. In this paper, we describe a new MIS-TLIF technique and pedicle screw insertion using a marker screw as a guidance method. Moreover, we report perioperative, postoperative, and patient-related outcomes. In addition, this paper outlines major differences in radiation exposure, cost effectiveness and accuracy of Marker Screw Minimally Invasive Transforaminal Interbody Fusion (MS-MIS TLIF) compared to other techniques. We report our technique to share our knowledge and experience with the aim of achieving a better MIS-TLIF that would help both surgeons and patients. *Materials and Methods*: A prospective case series was conducted between October 2018 and February 2021. Patients undergoing MS-MIS TLIF with marker screws were consecutively included. The surgery did not exceed two levels. The patients’ medical records were reviewed, and the included patients were asked to complete two outcome-questionnaires before surgery and at the six-month visit. The surgical technique is described in this paper. *Results*: A total of 37 patients were recruited. The mean age was 57.35 ± 12.8 years, and more than half of the patients were females. The most common indications for surgery were degenerative disc disease and spondylolisthesis, with the typical level at L4–5. The operative time was 3.02 ± 0.83 h, while the estimated blood loss was 127.7 ± 71.1 mL. The average time for ambulation and hospitalization was 1 ± 1.1 and 2.84 ± 1.4 days, respectively. The patients described significant improvement in both questionnaires. No screw-related complications or screw revisions were needed up to two years of follow-up. *Conclusions*: The use of marker screws for pedicle screw placement through a minimally invasive fashion is shown to be a promising technique that can overcome many drawbacks, including cost, operative time, and radiation exposure. Performing MS-MIS TLIF can achieve a 360- degree fusion compared to percutaneous MIS-TLIF.

## 1. Introduction

Transforaminal Lumbar Interbody Fusion (TLIF) has been both extensively reported and supported in the literature [1]. TLIF was first introduced to overcome the potential risk of nerve root injuries and dural tears associated with posterior lumbar interbody fusion [2]. TLIF can be achieved through open or minimally invasive surgery (MIS). During MIS-TLIF, the outcome of decompression and fusion appear to be similar to the open approach [1]. However, MIS-TLIF has evolved to decrease muscle dissection and operative morbidities associated with open surgery [1]. In MIS, a small incision provides access to the anterior column, which is aided by magnification devices such as loupes or a microscope [2]. However, there is no consensus in terms of how to define the technique. In one study, MIS-TLIF was found to yield better perioperative outcomes, early recovery, and decreased hospital stay [3]. On the other hand, radiation exposure was found to be doubled in a single-level MIS-TLIF when fluoroscopy was used vs. O-arm Navigation Systems [4].

In one study by Kim et al., the authors reviewed open TLIF in comparison to MIS-TLIF for short- and long-term outcomes. The operative time was reviewed in 16 studies for short-term outcomes. Two studies supported reduced operative time in MIS-TLIF, whereas nine studies supported open TLIF for shorter operative time, and five studies showed no difference [3]. Radiation exposure was also measured in both groups. The MIS-TLIF group was found to have higher radiation exposure, with 58 vs. 9 fluoroscopic shots measured as 1.9 mSv vs. 0.75 mSv in the MIS-TLIF and open TLIF group, respectively [3]. In addition, the MIS-TLIF group had a shorter time to ambulation and shorter hospital stay compared to the open group [3]. Kim et al. also compared the long-term outcomes after 12 months of follow-up between the two groups. Oswestry Disability Index (ODI) and Visual Analog Score (VAS) were studied in 12 articles. Half of the studies showed better improvement with the MIS-TLIF group; however, long-term follow-up at two years showed similar results between the two groups [3].

Economic studies on the cost effectiveness of open TLIF and MIS-TLIF were also reviewed. The direct and indirect costs were analyzed; however, no conclusive evidence was reported. Theoretically, the lower length of hospital stay and early return to work associated with MIS-TLIF can predict a lower cost. However, to the best of our knowledge, there is no strong evidence that precisely measures the cost effectiveness between these two surgical procedures [3]. Lastly, the Quality-Adjusted Life Years (QALYs) gained in both groups were similar at long-term follow-up [3].

Since the introduction of MIS-TLIF in the early 2000s, researchers have demonstrated decreased blood loss and lower rates of narcotic use [5]. Furthermore, its fusion rates were found to be similar to the open approach [5]. Many published articles have studied different techniques in regard to achieving fusion. Fujimori et al. compared posterolateral fusion (PLF) vs. TLIF and found TLIF to be superior in reducing the slippage and restoring disk height; however, similar fusion rates were reported [6]. Kim et al. studied fusion rates based on CT scan images by comparing PLF with TLIF vs. TLIF alone at 6 and 12 months post-operation. They found higher fusion rates in the PLF with TLIF group at both 6 and 12 months; in addition, the authors reported that adding TLIF would decrease the incidence of screw loosening [7]. Elmekaty et al. reported a better fusion rate favoring MIS-TLIF over Minimally Invasive Posterolateral Fusion (MIS-PLF) [8]. Moreover, there is a lack of evidence to compare MIS-TLIF vs. MIS-TLIF with added PLF. Theoretically, achieving both anterior and posterolateral fusions through MIS in one approach would be of great advantage, especially if they are performed simultaneously.

There is no consensus on the steps of MIS-TLIF as there are varieties of surgeons’ preferences discussed in the literature [5]. Researchers are eager to develop new techniques or modalities to increase pedicle screw placement’s accuracy and decrease radiation exposure. It has been shown that a mispositioned screw can lead to an increased hospital stay, chronic pain, or increased risk of deep infection [9]. Guidance methods vary, and new technologies has emerged to accurately place pedicle screws, such as O-arm Navigation, Robot-Assisted Surgery, and Augmented Reality Surgical Navigation (ARSN) [9]. Parker et al. studied conventional screw placement using fluoroscopy and found an intraoperative and postoperative revision of up to 4% for the screws inserted. In addition, an estimated cost per revision averaged to be more than USD 40,000 [10]. Using Robot-Assisted Surgery has been proven to increase the accuracy of pedicle screw placement and decrease perioperative complications [9]. However, the availability of these machines globally is challenging due to cost and learning curve difficulties.

In this paper, we describe our technique and experience with MIS-TLIF, utilizing marker screws as a guidance tool for pedicle screw insertion, given their clear advantages, availability, and lower radiation exposure in a minimal soft tissue dissection environment. Our technique targets 360-degree fusion surfaces. Moreover, we report the promising perioperative and postoperative outcomes achieved with the MS-MIS TLIF.

## 2. Materials and Methods

### 2.1. Study Participants

This study is a consecutive prospective case series, which was conducted between October 2018 and February 2021 at a tertiary hospital. Thirty-seven patients undergoing MS-MIS TLIF were consecutively selected and treated by the same surgeon. The operating surgeon had two spine fellowship trainings and more than 10 years in practice. Marker screws were used in all surgeries. Our inclusion criteria included all patients must be older than 18 years of age and were undergoing MS-MIS TLIF, and the surgery was planned for less than three levels. We excluded those who required fusion due to trauma, oncological and infectious causes. Patients with previous spinal surgery were also excluded.

### 2.2. Study Measures

The patients’ demographic data, comorbidities, and indications for surgery were reviewed. Perioperatively, data on the duration of surgery from incision to skin closure, estimated blood loss that could be visualized in suction canisters, surgical sponges, surgical field, and surgical levels involved were obtained. The collected postoperative variables included hemoglobin level, time before ambulation, and length of hospitalization. The patients were followed for 2 years and observed for any pedicle screw malpositioning, loosening, nonunion, or hardware-related complications.

The clinical outcomes were assessed using two self-administered questionnaires: the ODI and a 36-item short-form survey (SF-36). The ODI consists of 10 items, which can quantify disabilities related to lower back pain: the scores range from 0 to 100, with higher scores indicating more disability. The SF-36 involves 36 questions that cover 8 domains of health: physical functioning (PF), role limitations due to physical health (RP), role limitations due to emotional problems (RE), energy and fatigue (EF), emotional well-being (EW), social functioning (SF), body pain (BP), and general health (GH). Each item was scored from 0 to 100, with higher scores representing more favorable outcomes. SF-36 is also shown to be a valid tool for measuring outcomes after spine surgery [11]. A validated Arabic version of both questionnaires for the Saudi population was also used [12,13]. The questionnaires were completed by the patients before surgery and at the six-month follow-up visit, at which point any score changes were calculated. Two patients did not complete the questionnaires postoperatively, so they were not included in the analysis of the questionnaire data.

### 2.3. Surgical Intervention and Marker Screw Utilization

**Figure 1:**
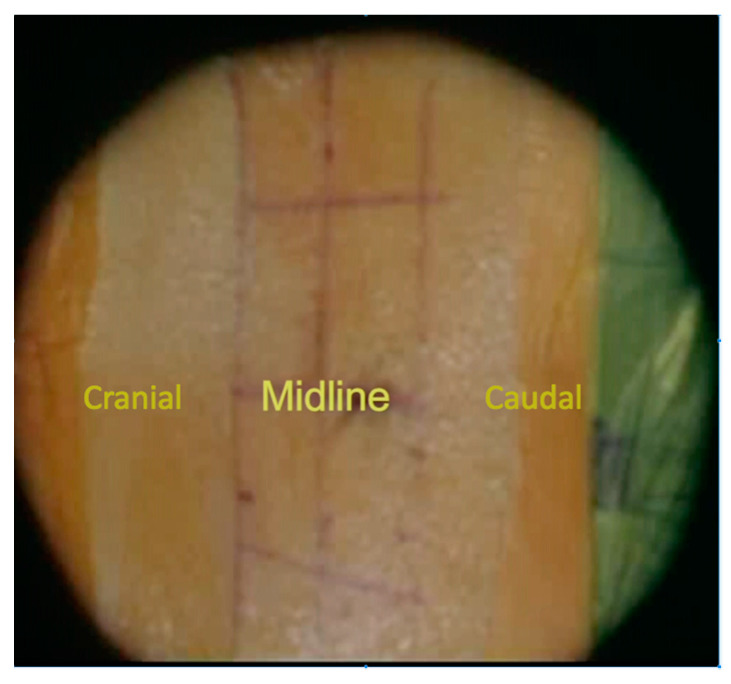
Skin marking is 1 cm lateral to the lateral pedicular wall (a single skin incision is utilized for adjacent levels).

**Figure 2:**
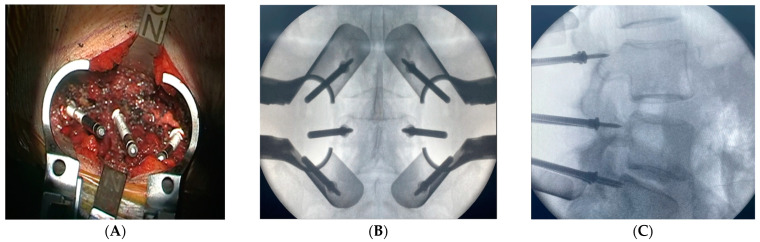
(**A**) Expandable retractor exposing the posterior elements and marker screws applied through standard anatomical entry point using a loupe or a microscope. (**B**,**C**) AP and lateral radiographs are taken after applying all marker screws to confirm the entry points and trajectories to start the insertion of pedicle screws.

**Figure 3:**
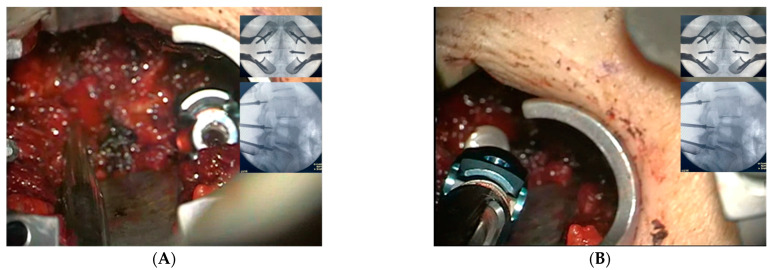
AP and lateral radiographs are saved in a fluoroscopic screen to aid the insertion of the pedicle screw utilizing the standard freehand technique. (**A**) Pedicle finder insertion using a trajectory based on the saved radiographs. (**B**) Pedicle screw insertion.

In a typical surgery, the patient is placed in a prone position. Using C-arm guidance, skin marking is made for minimally invasive access in a standard fashion for percutaneous pedicle screw insertion. The skin markings are located 1 cm lateral to the lateral pedicular wall, which are confirmed under the C-arm guidance, on each side for the planned levels. The skin incision is carried out with one vertical small incision instead of two—separated incisions per level (Figure 1). Sequential tubular dilators are subsequently used to develop the intermuscular approach, and then an expandable tubular retractor is applied. The involvement of a microscope/loupe is necessary throughout the procedure for proper visualization. The technique starts with the exposure of the posterior element to identify the anatomical landmarks for the application of the pedicle screws. This is accomplished by exposing the transverse process and pars interarticularis at the level intended for the pedicle screw insertion, which includes the facet joint, with preservation of the capsule without violation. Marker screws (Casper pins size 10 × 3.5) are inserted by creating a pilot hole at the anatomical entry point (the junctional point between the mid-transverse process and 3 mm lateral to the pars interarticularis) with a high-speed drill for all levels intended for the pedicle screw insertion (Figure 2A). Following the insertion of all marker screws, an AP and lateral X-rays are taken and saved on the screen to confirm the level and the trajectory (Figure 2B,C), thereby serving as a guide for the surgeon’s freehand technique with the pedicle screw insertion without the need for multiple shots of X-ray (Figure 3A,B).

**Figure 4:**
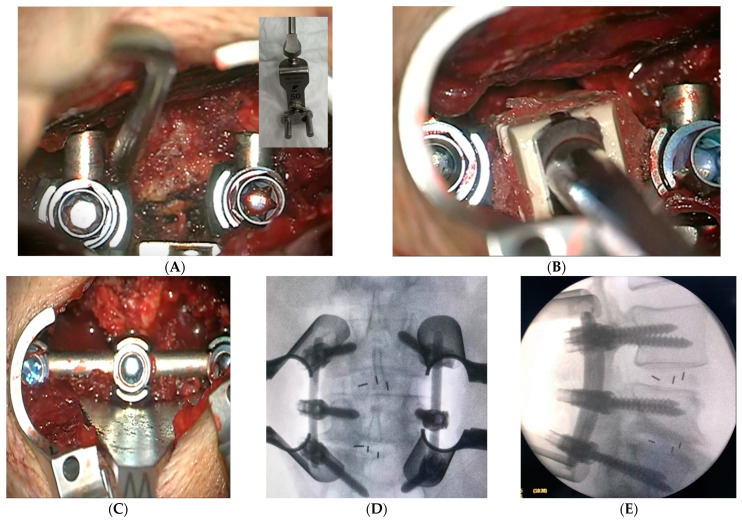
(**A**) Special retractor is applied to distract the two adjacent pedicle screws’ heads. (**B**) Following a sufficient decompression, the cage is then applied. (**C**) The rods are inserted, and the final tightening is carried out, along with posterolateral decortication and bone graft application. (**D**,**E**) AP and lateral radiographs are taken to confirm the pedicle screws and cage position.

**Figure 5:**
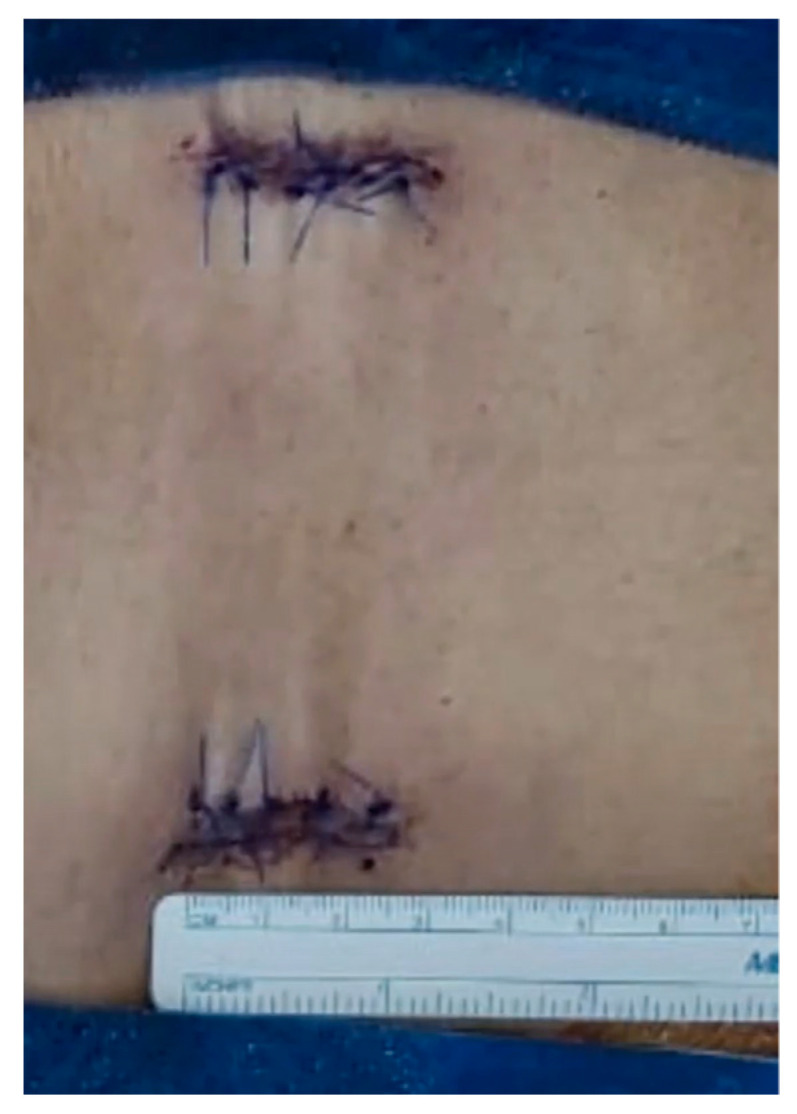
Closure of the fascia and subcutaneous skin in a standard fashion with less than 3 cm incision.

The transforaminal access is made between the pedicle screws’ heads, using a distractible retractor inserted over the head of the screws to allow space for foraminal access. A minimally invasive osteotome is used for partial facetectomy to further guide the access to the disk, in addition to collecting autogenous bone graft. Discectomy is performed with the preparation of the disc space for fusion and bone grafts, with an application of the interbody cage. X-ray is taken for sizing and accurate placement of the cage. Decortication of the posterolateral elements on both sides is made for 360-degree fusion. The rods are then inserted with the final tightening of all screws, followed by the application of allografts and final radiographs (Figure 4A–E). The tubular retractor is removed with the closure of the fascia and the subcutaneous layers of the skin in a standard fashion (Figure 5). For further demonstration, Supplementary Materials Video S1 shows the technique in a stepwise fashion.

### 2.4. Statistical Analyses and Ethical Considerations

Statistical analyses were performed using the IBM Statistical Package for Social Sciences Software for Windows, v. 25 (IBM Corp., Armonk, NY, USA). Descriptive analyses were also conducted, with the continuous variables being presented as mean ± standard deviations, while the categorical variables were presented as frequencies and percentages. The paired sample t-test determined significant changes in the ODI and SF-36 scores before and after surgery. A *p*-value less than 0.05 was considered significant. This study was approved by the Institutional Review Board of our institution. Data that could identify patient names and medical record numbers were not collected. Only the research authors had access to the study data. Informed consent was obtained from all participants.

## 3. Results

A total of 37 cases of MS-MIS TLIF were performed with marker screws. The gender distribution included 62.2% females and 37.8% males. The mean age at surgery was 57.92 ± 14.2 years, and the mean BMI was 32.8 ± 4.2 kg/m^2^. Patient comorbidities included diabetes mellitus in 11 cases (29.7%), hypertension in 15 cases (40.5%), chronic kidney disease in 2 cases (5.4%), dyslipidemia in 7 cases (18.9%) and coronary artery disease in 2 cases (5.4%). The patients’ demographics and comorbidities are summarized in Table 1.

The mean time since surgery was 28.4 ± 5.6 months. The most common indications for surgery were degenerative disc disease and spondylolisthesis, with 35.1% of patients for each indication, followed by foraminal stenosis in 16.2% of patients and prolapsed intervertebral disc in 13.5% of patients. Around half of the patients (45.9%) had surgery at the level of L4–L5. Two levels were involved in 32.4% of patients, followed by 16.2% of patients with surgeries at the level of L5-S1. In addition, 5.4% of patients had surgery at the level of L3–L4. The mean duration of surgery was 2.97 ± 0.7 h. While the estimated blood loss was 126.7 ± 72.1 mL, the decrease in hemoglobin was steady at 1.3 ± 0.6 g/dL. The time before ambulation was 1 ± 1.1 days, and the length of hospitalization post-surgery was found to be 2.68 ± 1.4 days. The surgical details are presented in Table 2. The Oswestry Disability Index scores were taken preoperatively and at a 6-month follow-up; the mean change difference was 29.1 ± 25.1 (mean ± SD). The SF-36 had the highest change in the domain of role limitations due to emotional problems, with a change difference of 47.7 ± 45.5 (mean ± SD), whereas emotional well-being had the lowest change, with a change difference of 18.3 ± 21.5 (mean ± SD). All patient scores on the ODI and SF-36 are illustrated in Table 3. The paired sample t-test showed significant improvement in the ODI and all eight domains of SF-36, (*p* < 0.001). Furthermore, the patients were followed up for 2 years postoperatively. During their follow-up, we analyzed each case for screw loosening or malposition, union, and hardware-related complications. We only encountered two complications in this series. The first case developed wound infection and underwent wound debridement. The second case developed proximal junctional kyphosis, and revision was performed for proximal extension.

## 4. Discussion

Pedicle screws are the mainstay of spinal fixation. Different approaches and guidance methods have been described in the literature, with variable advantages and disadvantages. Spine fixation systems have advanced with minimally invasive surgery to overcome complications. Guidance technologies have evolved from fluoroscopy to CT Navigation System and Robotic-Assisted Surgery for accurate screw placement. The published data on MIS-TLIF evaluate the efficiency of different techniques. Here, we discuss our technique to highlight its advantages and report our perioperative outcomes.

Open TLIF surgeries have been well described in the literature. MIS-TLIF was developed to improve patients’ recovery and decrease comorbidities. Emerging evidence comparing the two methods has shown better outcomes for MIS-TLIF. As these MIS techniques are performed more by surgeons, concerns have been raised regarding expected complications, and further studies are needed to develop new strategies to overcome these drawbacks. When it comes to conventional pedicle screw placement, surgeons performing an open TLIF will extensively dissect and expose soft tissues until the anatomical landmarks are identified and the pedicle screw entry points confirmed under fluoroscopy. Further radiographs are taken to place the pedicle screw in a proper trajectory with estimated nine shots of X-ray. In MIS-TLIF, the number of shots is even higher and reaches up to 58 shots as percutaneous insertion of the screw completely depends on radiographic visualization [3]. In our MS-MIS TLIF technique, we combined the advantages of both, namely minimal dissection and use of minimally invasive retractors, to identify anatomical landmarks and marker screws to help with trajectory, thereby saving the surgeon and patient from radiation exposure. The radiation hazard is negligible compared to other techniques as only AP and lateral radiographs are needed, which significantly reduces the patient and surgeon’s exposure to radiation. Nevertheless, the surgical team will acquire a safe distance during X-ray utilization.

There is no current consensus on how to define MIS-TLIF. Performing MIS-TLIF varies, depending primarily on a surgeon’s preference and their initial training. Multiple published articles have studied the outcome of these various techniques. Others have attempted to define and precisely describe MIS-TLIF to better understand the reported data on MIS-TLIF and to facilitate an accurate description of MIS-TLIF. Enriching the literature with surgeons’ experience from all over the world can help in reaching such a consensus. In a recent attempt to define MIS-TLIF using the existing literature, Lener et al. conducted a systematic review of 75 published articles concerning different TLIF techniques that included 7808 patients [5]. They concluded that the type of retractor is of paramount importance to defining MIS-TLIF [5]. Those with tubular expandable retractors are considered within the MIS-TLIF definition [5]. In our technique, we connect the two adjacent-level incisions in a similar way to the mini-open technique, although in a smaller fashion that dictates the use of tubular and expandible retractors. The length of the incision is almost equal to the sum of the two small percutaneous incisions. Favorably, with the help of these special MIS retractors, minimal soft tissue dissection is warranted, thus achieving the hallmark benefit of MIS-TLIF. The added benefit of our single incision MS-MIS TLIF is to increase the fusion surface by exposing the posterolateral surface for decortication and bone graft application. Moreover, using a single skin incision is advantageous and helpful to achieve posterolateral fusion that can reduce the risk of loosening and potentially increase the fusion rate [7]. A review of the literature shows that this is a novel technique for MIS-TLIF, and there are no comparative studies yet to analyze our MS-MIS TLIF to other described MIS-TLIF techniques.

Our series included 37 cases who underwent MS-MIS TLIF by the same surgeon, with marker screws being utilized in all surgeries to aid the entry points and screw trajectories. Our series show similar perioperative and postoperative outcome measures compared to those reported for common MIS-TLIF techniques [14,15,16]. Our mean time since surgery exceeds the two-year follow-up, which is comparable to those reported in the literature for observing complications. The main indications for surgical treatment in this series were degenerative disc disease and spondylolisthesis, mainly at the L4–5 level, which are similar to the included cases in other published articles. The mean duration of surgery, the estimated blood loss, and the decrease in hemoglobin postoperatively were all within the reported data from other MIS-TLIF publications. The post-operative measures, the time before ambulation, and the length of hospital stay were found to be minimal and comparable. These reported outcomes strengthen the existing MIS-TLIF advantages and quantify the outcomes of our technique. The post-operative functional scores based on the ODI and SF-36 were both statistically significant at the 6-month follow-up.

One of the major concerns in pedicle screw placement with the use of MIS-TLIF is radiation exposure for both the operating staff and patients. In a study by Rampersaud et al., spine surgeons were exposed to radiation 10 to 15 times greater than other musculoskeletal surgeons [17]. Additionally, minimally invasive spine surgeries are associated with significantly higher radiation exposure than open surgeries, as assessed by several studies [17,18,19]. The International Commission on Radiological Protection recommended a maximum yearly occupational exposure of less than 150 mSv to the lens, 500 mSv to the hand, and 20 mSv as an effective dose [20]. Concerning these guidelines, one study measuring radiation exposure during percutaneous pedicle screw fixation determined that the annual dose limit to the eye was reached after 645 procedures, while in another study, only 166 MIS-TLIF surgeries were enough to exceed the annual limit to the thyroid, if not protected [21,22]. Patients were exposed to radiation doses exceeding the annual recommended levels for non-radiation workers when considering their imaging prior to surgery, such as X-rays and computed tomography (CT), which account for 0.1 mSv and 6–7 mSv of exposure, respectively [23]. Several studies described methods and techniques to reduce radiation exposure. Marco et al. reported their data using a detachable pedicle marker and probe (DPMP) technique, along with a custom-designed probe to help decrease radiation and breach rate. They estimated an 80% reduction in the effective radiation dose with these techniques when compared to low-dose O-arm. However, the availability of such tools is challenging due to logistic reasons or institutional regulations for approval [24]. In another study, Kim et al. were able to decrease surgeons’ radiation exposure by 94.1% per screw by modifying the imaging technique and radiation source from real-time multiple-shot imaging; this was conducted with a continuous mode vs. intermittent single-shot imaging with a pulse mode [23]. In our MS-MIS TLIF technique, marker screws are utilized and inserted at all necessary levels to assess the entry points and guide the screw trajectories. Only two shots of fluoroscopy, namely AP and lateral, are taken to confirm the position, followed by pedicle screw insertion with a free-hand technique using the anatomical landmarks. This will give a great advantage by avoiding higher doses of radiation compared to percutaneous insertion, leading to efficient handling of instruments and faster time for insertion compared to conventional methods or Robot-Assisted Surgery.

Liu et al. compared the use of fluoroscopy and navigation in MIS-TLIF to increase pedicle insertion accuracy [24]. However, in their retrospective study, they found no statistical difference between MIS-TLIF with fluoroscopy or CT navigation guidance [24]. They also described technical challenges while utilizing the navigation system for the MIS technique due to a smaller incision size [24]. In one cadaveric study by Alhabib et al., conventional fluoroscopic-assisted and marker screw techniques were compared [25]. Breach was graded according to the Fu et al. grading system with the obtained CT images: the marker screw technique achieved the lowest breach rate in comparison to others [25]. In our series, marker screw utilization is considered to be a safe and reliable technique to identify entry points and trajectories. We did not find any complications associated with breach, and no screw revisions were needed.

Another concerning variable is operative time. For pedicle screw placement, Shin et al. studied the placement of 310 pedicle screws by using O-arm vs. conventional fluoroscopy [26]. They reported the mean preparation time for the O-arm-guided group reached 19 min vs. 4 min for the fluoroscopy-guided group, whereas the mean screw insertion time was 3.8 min for the O-arm group and 4.4 min for the fluoroscopy group [26]. Preparation for fluoroscopy is required only once with our MS-MIS-TLIF for single AP and lateral shots following the insertion of the marker screws; thus, we can achieve a shorter operative time.

The number of spinal instrumentation surgeries is expanding globally. The widespread of surgical interventions has accompanied an increase in health-related costs. In response, cost-effectiveness studies are attracting more attention in the comparison of various surgical techniques [27]. Furthermore, cost effectiveness cannot be overlooked and plays a major role in current healthcare systems. Previous economic studies on TLIF surgeries have failed to establish conclusive evidence [3]. However, in a recent published systematic review comparing open TLIF and MIS-TLIF, the authors concluded that MIS-TLIF might be more cost-effective than open TLIF [27]. The included articles are not of high-quality evidence, and further studies are warranted. Moreover, when comparing open TLIF to MIS, healthcare costs were higher in the MIS group by USD 26,526, and societal costs were lower in the MIS group by USD 36,927 [27]. Indeed, direct and indirect cost analysis is challenging in most reported articles. In our MS-MIS TLIF, we estimated an implant cost reduction approaching 60% for multiple reasons. First, the use of conventional screws in our technique, rather than MIS percutaneous screws, would potentially decrease the total implant cost. Second, the widespread availability and the non-disposal feature of marker screws as a guidance method would bring the total implant cost even lower. Third, comparably lower length of hospital stay and early return to work can further predict a lower cost, as supported by Kim et al. [3].

Fusion rates have also been studied by comparing different TLIF techniques. Fujimori et al. reported similar fusion rates between PLF and TLIF [6]. In another comparative study, Kim et al. studied posterolateral fusion when added to the TLIF in comparison to TLIF alone, and they found higher fusion rates in the TLIF-PLF group, in addition to a lower incidence of screw loosening [7]. In a study by Yao et al., the authors retrospectively analyzed fusion rates when comparing open vs. MIS TLIF [28]. They described a bone graft area ratio relative to the end plate surface area; furthermore, they divided the endplate into four quadrants. In both groups, the contralateral dorsal quadrant had a significantly lower bone graft area ratio and similar overall fusion rates [28]. However, an analysis of the non-union group showed a significant relation between the lower bone graft area ratio and 2-level surgery [28]. In MS-MIS TLIF, connecting the skin incisions for adjacent segments allows further posterolateral fusion surface and potentially increases fusion rates through a 360-degree fusion. This allows anterior and posterolateral fusion through minimally invasive access.

In our 37 case series using this novel MS-MIS TLIF, we found promising patient results, lower radiation exposure, more fusion surfaces, faster pedicle screw insertion and lower total implant cost, all through minimally invasive access. However, further studies are needed to directly compare this method to other MIS-TLIF techniques. Our study has a few limitations, including a small sample size, lack of randomization, lack of precise radiation exposure measurements, and lack of an accurate cost analysis. Further studies are needed to measure fusion rates and screws’ accuracy. Currently, we are conducting a comparative randomized study assessing the utilization of this novel MS-MIS TLIF.

## 5. Conclusions

Utilizing marker screws as a guidance method for pedicle screw placement can overcome many MIS-TLIF drawbacks, especially when it comes to radiation exposure and time efficiency for placing pedicle screws. Performing our novel MS-MIS TLIF technique would add the benefit of posterolateral fusion without the expense of invasive soft tissue dissection. From our experience, performing MS-MIS TLIF is safe and less expensive, provides 360-degree fusion, and is an available option with a significant reduction in radiation exposure than MIS-TLIF. However, more studies are needed with larger sample size, randomization, and precise radiation exposure calculations.

## Figures and Tables

**Table 1:** Patients’ Demographics and Comorbid Conditions.

Mean age (year)	57.92 ± 14.2
Gender (M/F)	14/23
Mean BMI (kg/m^2^)	32.8 ± 4.2
DM (%)	29.7
HTN (%)	40.5
CKD (%)	5.4
Dyslipidemia (%)	18.9
CAD (%)	5.4

DM: diabetes mellitus; HTN: hypertension; CKD: chronic kidney disease; CAD: coronary artery disease.

**Table 2:** Surgical Details and Immediate Post-operative Parameters.

The mean time since surgery (m)	28.4 ± 5.6
Indications (%)	
Degenerative disc disease	35.1
Spinal stenosis	16.2
Spondylolisthesis	35.1
Prolapsed intervertebral discs	13.5
Levels of surgery (%)	
L3–L4	5.4
L4–L5	45.9
L5–S1	16.2
Two levels	32.4
Duration of surgery (hours ± SD)	2.97 ± 0.7
Estimated blood loss (mL ± SD)	126.7 ± 72.1
Decrease in hemoglobin (g/dL ± SD)	1.3 ± 0.6
Hospitalization time (days ± SD)	2.68 ± 1.4
Time before ambulation (days ± SD)	1 ± 1.1

**Table 3:** Mean patients’ scores on the ODI and SF-36 (*n*: 35).

	Preoperative (Mean ± SD)	Six Months Postoperative (Mean ± SD)	*Improvement in Score* (Mean ± SD)	*p-Value*
**ODI**	60.3 ± 17.5	28.6 ± 18.9	29.1 ± 25.1	0.000 *
**SF-36**				
Physical functioning	25 ± 23.7	51.5 ± 29.1	27.4 ± 39.3	0.000 *
Role limitations due to physical health	12.8 ± 32.8	52.1 ± 42.6	39.8 ± 41	0.000 *
Role limitations due to emotional problems	20.9 ± 37.9	68.5 ± 40.3	47.7 ± 45.5	0.000 *
Energy and fatigue	30 ± 18.6	58.4 ± 20.1	28.1 ± 25.9	0.000 *
Emotional well-being	49.7.3 ± 14.7	67.6 ± 17.3	18.3 ± 21.5	0.000 *
Social functioning	40.3 ± 28.1	74.2 ± 28.5	34.1 ± 37.9	0.000 *
Body pain	26.3 ± 24.4	64.5 ± 25.8	37.3 ± 34.6	0.000 *
General health	44.5 ± 19.8	65.2 ± 17	20.8 ± 21.8	0.000 *

ODI: Oswestry Disability Index, SF-36: 36-item short form survey, SD: standard deviation, * *p* < 0.01.

## Data Availability

All obtained patients data were gathered by the authors and coded to maintain privacy and confidentiality of all included, only authors hade access to the collected data.

## Supplementary Materials

The following supporting information can be downloaded at https://www.mdpi.com/article/10.3390/medicina59030585/s1. Video S1: Demonstration of MIS-TLIF technique using marker screw as a guidance method.
